# Supplementary material for: Extraction, Identification, and Quantification of Polyphenols from the Theobroma cacao L. Fruit: Yield vs. Environmental Friendliness
Source: Foods. 2024 Jul 29;13(15):2397. doi: 10.3390/foods13152397 (PMC11312112; doi:10.3390/foods13152397)
Supplement: Supplementary file 1 [file foods-13-02397-s001.zip › foods-3081591-supplementary.pdf]

Supplementary Material

Extraction, Identification, and Quantification of Polyphenols from the Theobroma cacao L. fruit: Performance vs. Environmental Friendliness

Juan Manuel Silva[1,2], Fernanda Peyronel[2*], Yinan Huang[2], Carlos Boschetti[1], Maria G. Corradini[2,3*]

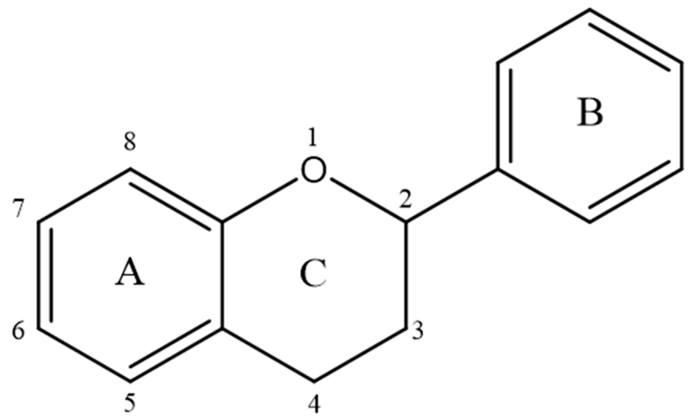

Supplementary Figure S1A. Schematic view of the flavonoid basic structure

<table>
<tr><th colspan="2">Main Structure/Backbone</th></tr>
<tr><td colspan="2">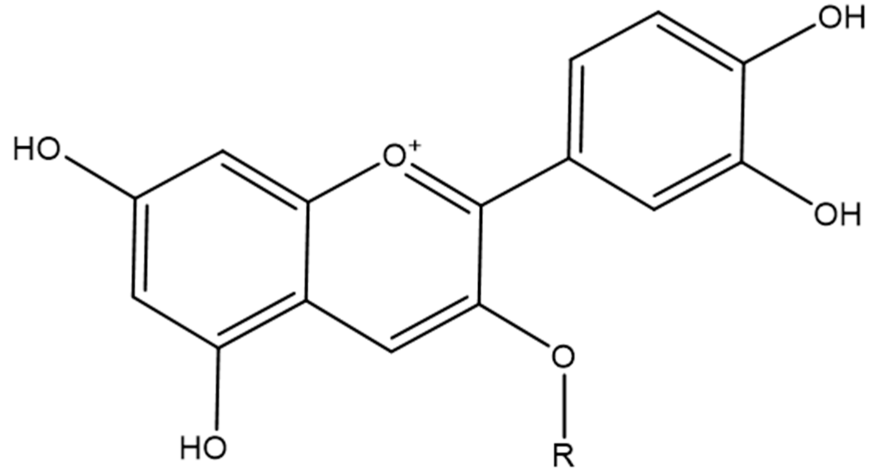
</td></tr>
<tr><th>Glycosyl group (-R)</th><th>Compound Name</th></tr>
<tr><td>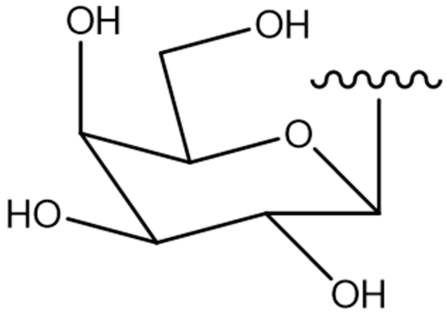
</td><td>Cyanidin-3-O-galactoside</td></tr>
<tr><td>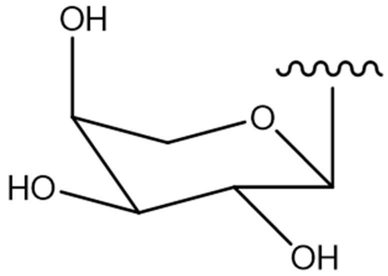
</td><td>Cyanidin-3-O-arabinoside</td></tr>
</table>

Supplementary Figure S1B. Main anthocyanins found in cacao.

Main Structure/Backbone	
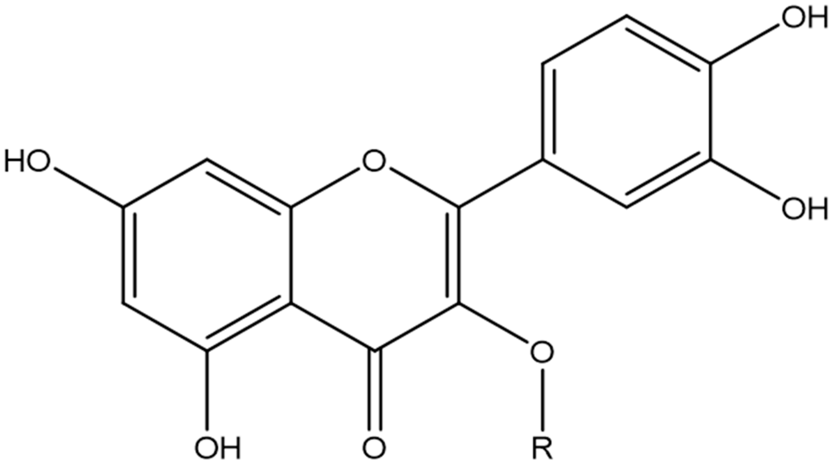	
**Glycosyl group (-R)**	**Compound Name**
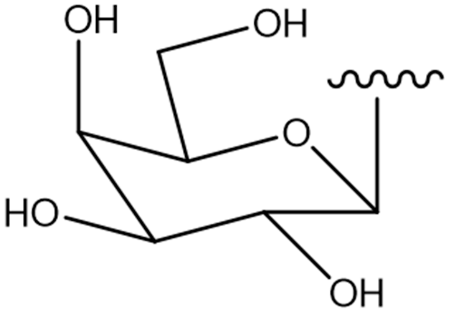	**Quercetin-3-O-galactoside**
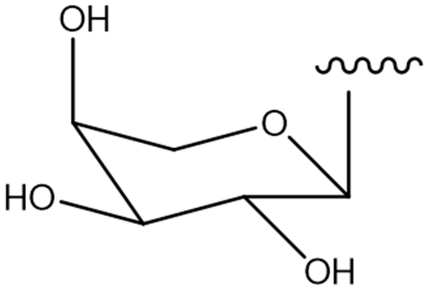	**Quercetin-3-O-arabinoside**
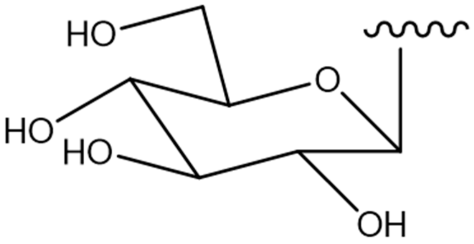	**Quercetin-3-O-glucoside**

Supplementary Figure S1C Commonly found flavonols in cacao.

Structural formulas - *Flavanols monomers*	Compound Name
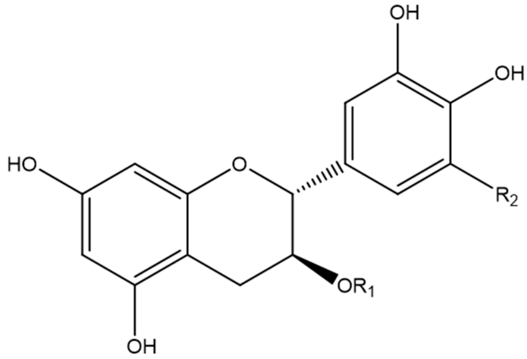	R_1=H; R_2=H (+)Catechin<br>R_1=H; R_2=OH (+)Gallocatechin
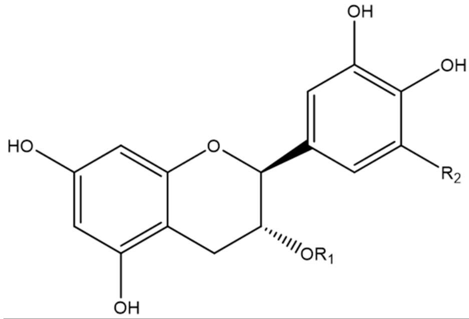	R_1=H; R_2=H (-)Catechin<br>R_1=H; R_2=OH (-)Gallocatechin
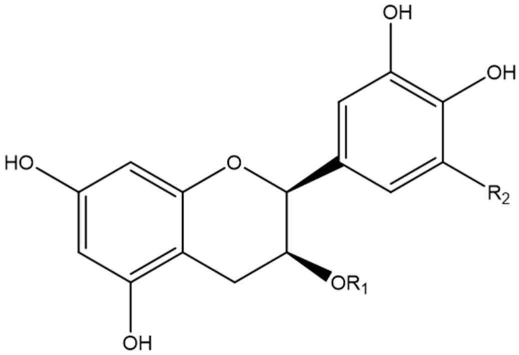	R_1=H; R_2=H (+)Epicatechin<br>R_1=H; R_2=OH (+)Epigallocatechin
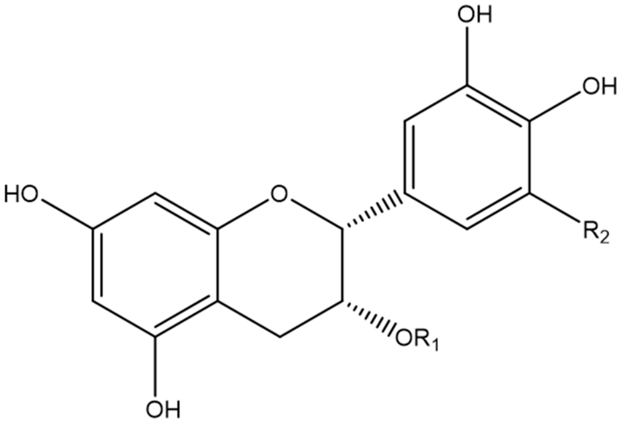	R_1=H; R_2=H (-)Epicatechin<br>R_1=H; R_2=OH (-)Epigallocatechin

Supplementary Figure S1D. Common flavanols found in cacao.

<table>
<tr><th colspan="2">Main Structure/Backbone</th></tr>
<tr><td colspan="2">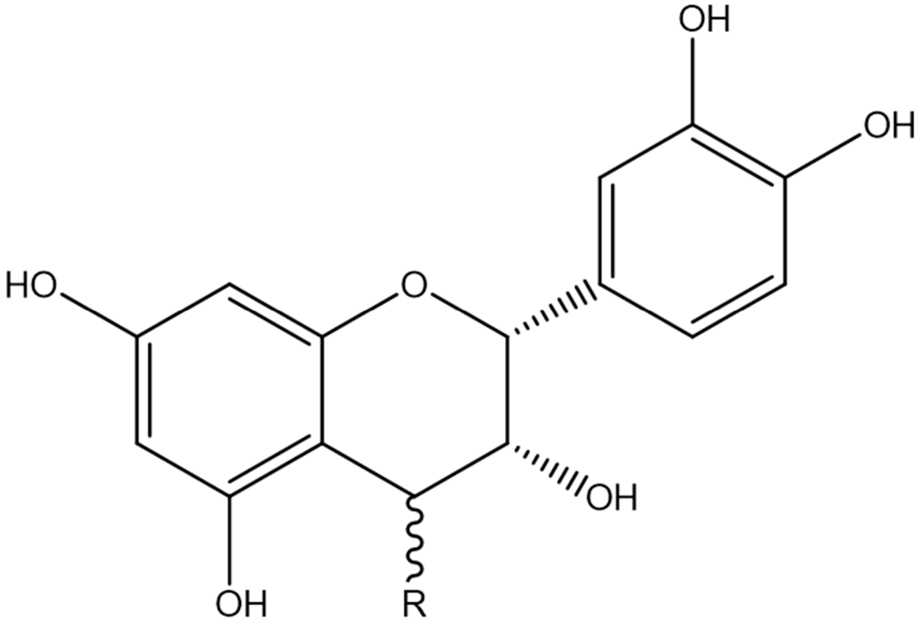
</td></tr>
<tr><th>Substitution monomer (-R)</th><th>Compound Name</th></tr>
<tr><td>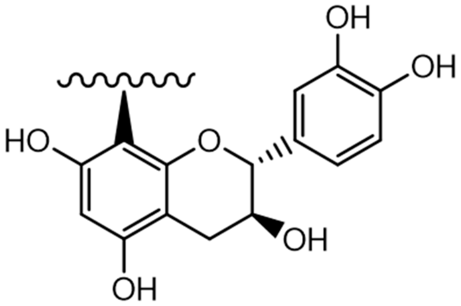
</td><td>Procyanidin B1</td></tr>
<tr><td>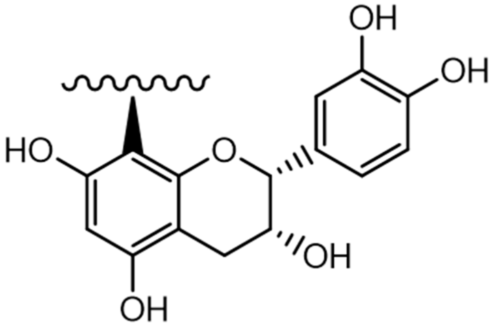
</td><td>Procyanidin B2 dimer</td></tr>
</table>

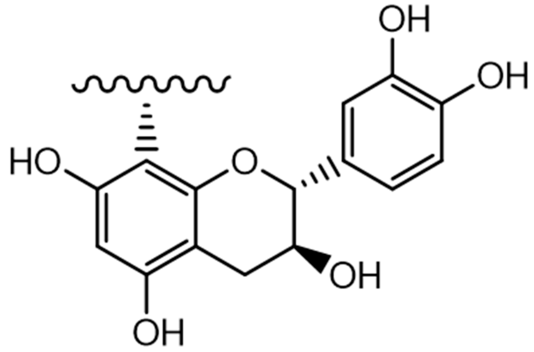	**Procyanidin B4 dimer**
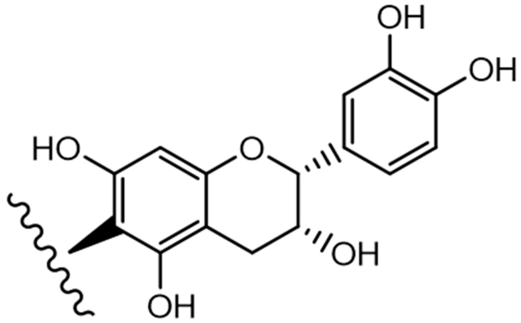	**Procyanidin B5 dimer**
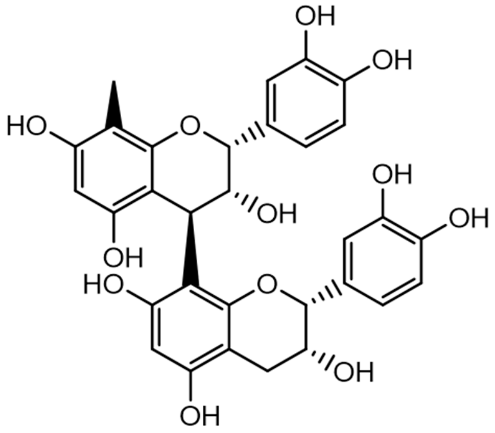	**Procyanidin C1 dimer**

Supplementary Figure S1E. Type B Procyanidins commonly found in cacao.

Main Structure/Backbone	
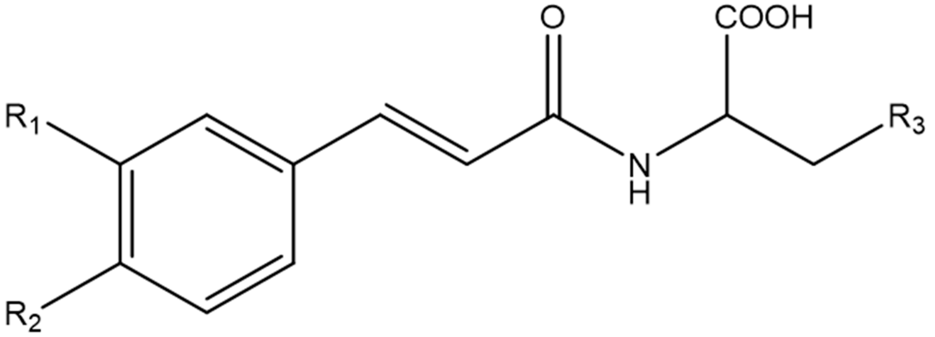	
**Functional groups and their position**	**Compound Name**
**R_1=OH; R_2=OH; R_3=** 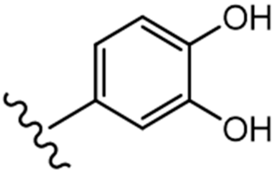	**N-Caffeoyl-3-O-hydroxytyrosine**
**R_1=H; R_2=OH; R_3=** 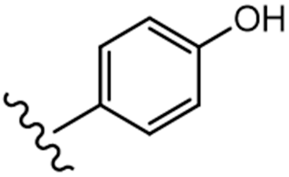	**N-p-Coumaroyl-tyrosine**
**R_1=H; R_2=H; R_3= COOH**	**Cinnamyol-L- aspartic acid**
**R_1=OH; R_2=OH; R_3= CH_2COOH**	**Caffeoyl-L-glutamic acid**

Supplementary Figure S1F. Structural formulas of phenolic acids commonly found in cacao.

Supplementary Table S1. Reagent/Solvents reported and their penalty points calculated as proposed by the Eco-Scale in which the PP from the pictogram are multiplied by the PP attributed to the hazard word.

CAS#	Reagent/Solvent name and chemical formula		PP due to pictogram	PP due to hazard word	Reagent/ Solvent Risk PP
67-63-0	2-propanol	$(CH_3)_2CHOH$	2	2	4
128446-34-4	(2-Hydroxypropyl)-γ-cyclodextrin	$C_{72}H_{128}O_{48}$	1	1	2
6203-18-5	4-(dimethylamino) cinnamaldehyde	4-$[(CH_3)_2N]C_6H_4CH{=}CHCHO$	1	1	2
64-19-7	Acetic acid	CH_3CO_2H	2	2	4
67-64-1	Acetone	CH_3COCH_3	2	2	4
1975-05-08	Acetonitrile	CH_3CN	4	2	6
7446-70-0	Aluminum chloride	$AlCl_3$	2	2	4
540-69-2	Ammonium formate	HCO_2NH_4	1	1	2
7783-83-7	Ammonium iron(III) sulfate dodecahydrate	$(NH_4)Fe(SO_4)_2 \cdot 12H_2O$	1	1	2
7783-83-7	Ammonium iron(III) sulfate dodecahydrate	$NH_4Fe(SO_4)_2$:12H2O	No data	No data	0
7440-37-1	Argon	Ar	1	1	2
71-36-3	Butanol	$CH_3(CH_2)_3OH$	2	1	3
124-38-9	Carbon dioxide	CO_2	1	2	3
154-23-4	Catechin	$C_{15}H_{14}O_6$	1	1	2
528-58-5	Cyanidin chloride	$C_{15}H_{11}ClO_6$	0	0	0
91053-39-3	Diatomaceous earth	Si O_2	1	0	1
1975-09-02	Dichloromethane	CH_2Cl_2	3	1	4
7732-18-5	Dihydrogen oxide/ water	H_2O	0	0	0
75-18-3	Dimethyl sulfide	$(CH_3)_2S$	1	1	2
67-68-5	Dimethyl sulfoxide	$(CH_3)_2SO$	1	1	2

490-46-0	Epicatechin	$C_{15}H_{14}O_6$	1	1	2
64-17-5	Ethanol	CH_3CH_2OH	2	2	4
141-78-6	Ethyl-acetate	$CH_3COOC_2H_5$	2	2	4
*	Folin-Ciocalteu Phenol reagent		1	2	3
64-18-6	Formic acid	$HCOOH$	2	2	4
149-91-7	Gallic acid	$(HO)_3C_6H_2CO_2H$	0	1	1
7440-59-7	Helium	He	1	1	2
110-54-3	Hexane	$CH_3(CH_2)_4CH_3$	4	2	6
7647-01-0	Hydrochloric acid	HCl	2	2	4
27661-36-5	Idaein chloride	$C_{21}H_{21}ClO_{11}$	No data	No data	0
67-56-1	Methanol	CH_3OH	3	2	5
142-82-5	N-heptane/normal heptane	$CH_3(CH_2)_5CH_3$	4	2	6
110-54-3	N-hexane/ normal hexane	$CH_3(CH_2)_4CH_3$	3	2	5
7727-37-9	Nitrogen	N_2	1	1	2
8032-32-4	Petroleum ether	C_6H_{14}	2	2	4
7664-38-2	Phosphoric acid	H_3PO_4	1	2	3
153-18-4	Rutin	$C_{27}H_{30}O_{16}$	1	1	2
9041-37-6	Sephadex LH-20/ lipophilic sephadex	$C_9H_8N_2O_3S$	No data	No data	0
144-55-8	Sodium bicarbonate	$NaHCO_3$	1	1	2
**	Sodium borate buffer solution		1	2	3
497-19-8	Sodium carbonate	Na_2CO_3	1	1	2
141-53-7	Sodium formate	$CHNaO_2$	1	0	1
1310-73-2	Sodium hydroxide	$NaOH$	2	2	4
7632-00-0	Sodium nitrite	$NaNO_2$	3	2	5
7757-82-6	Sodium sulfate	Na_2SO_4	1	1	2

1976-03-09	Trichloroacetic acid	$C_2HCl_3O_2$	2	2	4
1976-05-01	Trifluoroacetic acid	CF_3COOH	2	2	4
53188-07-1	Trolox	$C_{14}H_{18}O_4$	1	1	2
*	10377-48-7/13472-45-2/7664-38-2/7647-01-01/7631-95-0				
**	7732-18-5/1303-96-4/1310-73-2				

Information was obtained from vendors' websites.

Supplementary Table S2. Equipment reported, showing kWh usage for the listed working conditions from which the energy consumption penalty points (PP) were calculated using the criteria in Table 2.

Equipment	Conditions		Kilowatt per hour (kWh)	PP
	Temperature (ºC)	Time		
**Automatic sieve**			<0.1	0
**Blender**		≤20 min	<0.1	0
**Centrifugal partition chromatography (SCPC)**			>1.5	2
**Centrifugation**		≤60 min	≤1.5	1
	4		>1.5	2
**Dry ice/ liquid nitrogen**			≤1.5	1
**Drying (air/ hot air)**			<0.1	0
		48 h	>1.5	2
**Electric oven**		>24h	>1.5	2
**Electronic tongue technique**			>1.5	2
**Freeze dry**			≤1.5	1
**Gel permeation chromatography**			>1.5	2
**Grinder**			<0.1	0
**Heater**	50	<1 h	<0.1	0
	50	7 h	>1.5	2
**Hot water extraction equipment**			>1.5	2
**HPLC (DAD, FLD, TOF, MS, ESL, UV, FLPC, ECD, CAD, PDA)**			>1.5	2
**Hydrodynamic cavitation**			>1.5	2
**Incubator**	60	<45 min	<0.1	0
	70	74 min	≤1.5	1
	45	120 min	≤1.5	1
		6 d	>1.5	2
**Incubator + shaker**	35	30 min	<0.1	0
	95		≤1.5	1
		>1h	≤1.5	1
**Incubator +vacuum**	<55		≤1.5	1
**lyophilization**			>1.5	2
**Micro plate reader**			<0.1	0
**Microwave dry**			≤1.5	1
**Mill (knife, ball)**			<0.1	0
**Mixer**			<0.1	0
**Near Infrared Spectrophotometer (NIR)**			>1.5	2

**Orbital shaker**			<0.1	0
**Oven / electric oven**	40	20min	<0.1	0
	110	>24 h	>1.5	2
**PEF**			≤1.5	1
**Presser**			≤1.5	1
**Pressurized liquid Extraction (ple)**			>1.5	2
**Refrigeration**	> 4	Overnight	≤1.5	1
	< -4		>1.5	2
**Roaster**			≤1.5	1
**Rotary evaporator**	40		≤1.5	1
		>1h	>1.5	2
**Shaker**		6 h	<0.1	0
**Spectrophotometer**			≤1.5	1
**Supercritical fluid extraction (SFE)**			>1.5	2
**Ultra-centrifugal mill**			≤1.5	1
**Ultrasonic bath**		≤60min	<0.1	0
	50	>60 min	≤1.5	1
**Ultra-turrax**			≤1.5	1
**UPHLC - MS**			>1.5	2
**Vacuum drying**	40		≤1.5	1
**Vortex**			<0.1	0
**Water bath**	70		<0.1	0
